# Telehealth Competencies for Interprofessional Teams Caring for Older Adults and Care Partners

**DOI:** 10.1093/geroni/igaa057.2886

**Published:** 2020-12-16

**Authors:** Becky Powers, Kathryn Nearing, Studi Dang, William Hung, Hillary Lum

**Affiliations:** 1 South Texas Veterans Health Care System, San Antonio, Texas, United States; 2 University of Colorado Anschutz Medical Campus, Aurora, Colorado, United States; 3 Miami GRECC, Miami, Florida, United States; 4 Icahn School of Medicine at Mount Sinai; James J Peters VA Medical Center, Bronx, New York, United States; 5 VA Eastern Colorado GRECC, Aurora, Colorado, United States

## Abstract

Providing interprofessional geriatric care via telehealth is a unique clinical skillset that differs from providing face-to-face care. The lack of clear guidance on telehealth best practices for providing care to older adults and their care partners has created a systems-based practice educational gap. For several years, GRECC Connect has provided interprofessional telehealth visits to older adults, frequently training interprofessional learners in the process. Using our interprofessional telehealth expertise, the GRECC Connect Education Workgroup created telehealth competencies for the delivery of care to older adults and care partners for interprofessional learners. Competencies incorporate key telehealth, interprofessional and geriatric domains, and were informed by diverse stakeholders within the Veterans Health Administration. During this symposium, comments will be solicited from attendees. Once finalized, these competencies will drive the development of robust curricula and evaluation measures aimed at training the next generation of interprofessional providers to expertly care for older adults via telehealth.

